# A Review on Pharmacological Properties of Zingerone (4-(4-Hydroxy-3-methoxyphenyl)-2-butanone)

**DOI:** 10.1155/2015/816364

**Published:** 2015-05-27

**Authors:** Bilal Ahmad, Muneeb U. Rehman, Insha Amin, Ahmad Arif, Saiema Rasool, Showkat Ahmad Bhat, Insha Afzal, Ishraq Hussain, Sheikh Bilal, Manzoor ur Rahman Mir

**Affiliations:** ^1^Molecular Biology Lab, Division of Veterinary Biochemistry, Faculty of Veterinary Sciences & Animal Husbandry, Sher-e-Kashmir University of Agricultural Science & Technology (SKUAST-K), Srinagar, Jammu and Kashmir 190006, India; ^2^Forest Biotech Lab, Department of Forest Management, Faculty of Forestry, Universiti Putra Malaysia, 43400 Serdang, Selangor, Malaysia; ^3^Division of Livestock Production & Management, Faculty of Veterinary Sciences & Animal Husbandry, Sher-e-Kashmir University of Agricultural Science & Technology (SKUAST-K), Srinagar, Jammu and Kashmir 190006, India

## Abstract

Humans have been using natural products for medicinal use for ages. Natural products of therapeutic importance are compounds derived from plants, animals, or any microorganism. Ginger is also one of the most commonly used condiments and a natural drug in vogue. It is a traditional medicine, having some active ingredients used for the treatment of numerous diseases. During recent research on ginger, various ingredients like zingerone, shogaol, and paradol have been obtained from it. Zingerone (4-(4-hydroxy-3-methoxyphenyl)-2-butanone) is a nontoxic and inexpensive compound with varied pharmacological activities. It is the least pungent component of *Zingiber officinale*. Zingerone is absent in fresh ginger but cooking or heating transforms gingerol to zingerone. Zingerone closely related to vanillin from vanilla and eugenol from clove. Zingerone has potent anti-inflammatory, antidiabetic, antilipolytic, antidiarrhoeic, antispasmodic, and so forth properties. Besides, it displays the property of enhancing growth and immune stimulation. It behaves as appetite stimulant, anxiolytic, antithrombotic, radiation protective, and antimicrobial. Also, it inhibits the reactive nitrogen species which are important in causing Alzheimer's disease and many other disorders. This review is written to shed light on the various pharmacological properties of zingerone and its role in alleviating numerous human and animal diseases.

## 1. Introduction 

Ginger (*Zingiber officinale *Roscoe, family: Zingiberaceae) originated in South-East Asia and is the most common spice, used all over the world. It is a pungent, aromatic spice which adds a special flavor and zest to our food. Ginger is the underground rhizome of the ginger plant. This perennial herb is largely grown as both a spice and a condiment. Besides, being used to give beautiful aroma to Indian food [1], ginger is also used as a potent medicine. Ginger holds a profound mention in ancient Chinese, Indian, and Middle Eastern writings. It has always been prized for its aroma, culinary, and, above all, distinct medicinal properties.

Commonly, ginger is not found to be an allergenic food as such. It is widely known to treat a number of different diseases throughout the world. It is due to varied phytochemistry of ginger that it has large health benefits. It contains various minerals and vitamins as well as enzymes like zingibain, a proteolytic enzyme. Ginger contains numerous active compounds which vary significantly between plant varieties and regions in which it is grown. More than 60 active constituents are known to be present in ginger, which have been broadly divided into volatile and nonvolatile compounds. Hydrocarbons mostly monoterpenoid hydrocarbons and sesquiterpene include the volatile component of ginger and impart distinct aroma and taste to ginger. On the other hand, nonvolatile compounds include gingerols, shogaols, paradols, and also zingerone. Zingerone is produced during drying of ginger directly and also by thermal degradation of gingerols or shogaols.

Zingerone is present in a significant amount of about 9.25% in ginger. It is a member of Methoxyphenol family and its related derivatives. They have a basic phenolic ring with a methoxy group attached to benzene ring. Zingerone is known to have potent pharmacological activities. Zingerone is primarily present in dry ginger, but cooking or drying also converts gingerol (another component in ginger) into zingerone by retroaldol reaction [2]. Use of high profile liquid chromatography has shown that the contents of 6-gingerol, 8-gingerol, and 10-gingerol are usually low in fresh ginger while on drying and roasting the amount of zingerone increases significantly [2].

**Figure 1:**
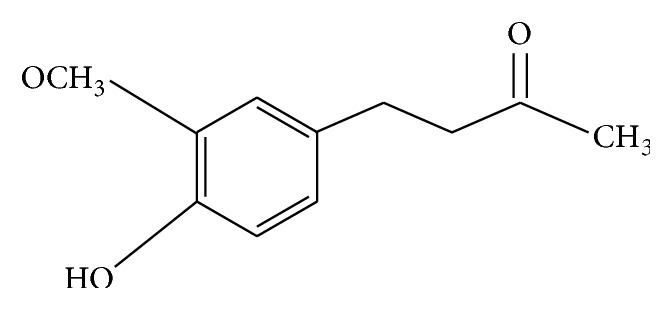
Chemical structure of zingerone. IUPAC name: [4-(3-methoxy-4-hydroxyphenyl)-butan-2-one].

**Figure 2:**
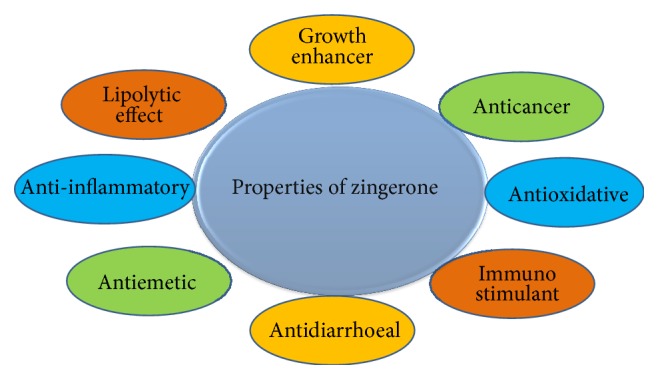
Pharmacological dimensions of zingerone.

In the year 1945, Cotton was the first to patent the manufacturing process for zingerone synthesis [3]. Chemically, zingerone is vanillyl acetone which is a member of phenolic alkanone group [4] (see Figure 1). Its pharmacological properties are varied (see Figure 2) including antioxidant [5], anti-inflammatory [6], anticancer [7], and antimicrobial activities [8]. Pharmacokinetics of zingerone has revealed that administration of zingerone either orally or intraperitoneally results in oxidation of side chain at all available sites. During catabolism of zingerone, glucuronidation and sulfation occur which leads to excretion of glucuronide compounds and sulphate conjugates in urine within 24 hours of consumption. Therefore, ginger has many medicinal properties besides being highly used as a flavouring agent all over the world. It can be widely used in alleviating many diseases of humans and animals.

The therapeutic potential of zingerone has been discussed under the following headings.

## 2. Zingerone as Antioxidant and Anti-Inflammatory

Ginger can be regarded as the storehouse of antioxidants. It has an extraordinary property of scavenging reactive oxygen species (ROS), free radicals, peroxides, and various other damaging oxidants. The active ingredients like gingerols, shogaols, zingerone, and so forth present in ginger exhibit antioxidant activity. It inhibits an enzyme, namely, xanthine oxidase, which is mainly involved in the generation of reactive oxygen species. The first evidence suggestive of antioxidant properties exhibited by zingerone is that zingerone has the ability to degrade free radicals generated by radiolysis of various food products. The observation that zingerone minimizes oxidation of lipids undoubtedly signifies its role as an antioxidant. It was supported by the fact that zingerone suppresses ferric ascorbate induced lipid peroxidation in rat brain [9]. Zingerone has been reported to protect in vitro DNA against stannous chloride induced ROS oxidative damage [5]. Zingerone provides direct adaptogenic effect by preventing oxidative stress on smooth muscles of intestine [10]. Further, zingerone administration was found to reduce the mitochondrial injury and peroxidation of lipids and downregulate some proapoptotic proteins like Bax, Apaf-1, and Caspases 3–9 [11]. These findings lead to a conclusion that zingerone is a potent antioxidant.

Another exciting fact regarding zingerone is that it has high antioxidant activity in comparison to ascorbic acid [5]. Another study revealed that zingerone has scavenging effect against peroxynitrite formed from the reaction of superoxide and nitric oxide inducing cellular and tissue damage [12]. Recently, a study found that zingerone by virtue of its antioxidant activity protects the heart of rats against the isoproterenol induced myocardial infarction [13]. Similarly the anti-inflammatory activities of ginger and its active compounds have been revealed by various workers [14].

Oxidation is the main cause for the pathogenesis of various inflammatory conditions including cancers, neuronal cell injury, hemorrhage, and septic shock [15]. Till today, no safe therapeutic drug for the treatment of inflammatory conditions is present as such. Zingerone treatment has potential of being used as a potent drug for treatment of diseases like Alzheimer's and atherosclerosis. Again, this property owes to its ability to scavenge free radicals which have oxidative properties. Thus, zingerone can inhibit reactive oxygen species (ROS) and maintain its antioxidant properties. So, the treatment with zingerone in these patients might prove to be beneficial [11]. One of the important neuronal diseases called Parkinson's disease can be prevented by the use of this potent by-product of ginger, due to its leading antioxidant properties of degrading free radicals and superoxide ions [16]. This was elucidated when zingerone was known to reduce the striatal dopamine formation and its various metabolites [17].

More studies were done to observe the antioxidant properties of zingerone by studying its effect on PPAR and NF-*κ*B. Zingerone was found to suppress activity of both PPAR and NF-*κ*B [18]. The other evidence for the alteration of NF-*κ*B activity by zingerone was provided in a study that suggested the zingerone inhibits colitis in rats by downregulating NF-*κ*B activity and IL1*β* signaling pathway [19]. Recently, molecular mechanisms of zingerone treatment demonstrated that zingerone inhibits age-dependent proinflammatory NF-*κ*B expression and interferes with MAPK signaling pathway [6].

A recent study revealed anti-inflammatory nature of zingerone against LPS induced hepatic injury/inflammation in mice [20]. Thus, zingerone was found to act as a hepatoprotective agent as a result of its ability to scavenge free radicals and cause suppression of inflammatory mediators in mice. With this many areas for future research and development opened up. Thus, the combined, potent anti-inflammatory and antioxidant activity of zingerone can prove to be a remedial measure for prevention and treatment of a number of varied diseases including cancer, diabetes, cardiovascular disease, arthritis, and osteoporosis.

## 3. Zingerone as Antidiarrhoeic

Enteric infections are havoc to the whole world. They badly affect both human and livestock populations all over the globe. Acute diarrhea is a leading cause for mortality in both young and adult individuals mostly due to high loss of fluids from a diarrhoeic patient. While acute diarrhoea is a major cause of childhood mortality in developing countries, mortality of adults takes a toll particularly during epidemics of diarrhoea. The mechanisms involved in diarrhea include osmotic disturbances, secretory abnormalities, inflammation of intestines and abnormalities of intestinal motility. The continued prevalence of acute diarrhoea in children and adults is due to consumption of contaminated food and water together with unhygienic eating habits.* E. coli* is found to be a potent cause of enteric infections and diarrhoea. It is known to release enterotoxins which inhibit motility of digestive tract like colon part of intestines. Studies have shown that zingerone has the ability to inhibit enterotoxins of various pathotypes of* E. coli* induced fluid secretion in the ileum of mice [21] and inhibits colonic motility not only in vitro but also in vivo in rats [22]. Since abnormal facilitation of gastrointestinal motility and excessive fluid secretion of gastrointestinal tract cause diarrhoea, zingerone is likely the active principle responsible for the antidiarrhoeal activity of ginger. Some natural pungent compounds such as capsaicin activate a nonselective cation channel termed transient potential vanilloid-1 (TRPV1). Also, zingerone has been demonstrated to evoke opening of TRPV1 [23]. Zingerone, a pungent analogue of ginger, exerts an inhibitory effect on colonic motility by directly acting on smooth muscles of colon [22]. Zingerone is responsible for antidiarrhoeal activity of ginger and it modifies bacterial as well as host cell metabolism [21].

## 4. Zingerone Antagonizes Radiation-Induced Stress

Nowadays, radiation therapy, in the form of X-rays, gamma rays, infrared rays, and so forth, is being used in patients suffering from various severe diseases including treatment of cancer [24]. Patients are also being exposed to radiations during diagnosis of various orthopedic, gynecological, cardiological, and respiratory disorders. There are drastic drawbacks of using radiations on the body. Radiation therapy leads to oxidative stress in organs which are exposed to radiations, rendering present available radioactive compounds harmful and toxic even at optimal concentration. Basically, there is again an oxidative stress induced by radiations. Radiations cause the formation of reactive oxygen species (ROS) which damage the organs and induce stress in the patient. Documented by many researchers, zingerone has been found to neutralize radiation-induced oxidative free radicals [25].

It has also been proved that zingerone exerts several biological effects, by inhibiting the mutation in* E. coli* induced by ultraviolet (UV) radiation [26], superoxide anions, and peroxynitrate radicals in vitro [12]. Zingerone also exhibits potent radioprotective effects against radiation-induced toxicity in Swiss Albino mice [25]. The possible mechanism responsible for zingerone's radioprotective effect is that zingerone neutralizes radiation-induced reactive oxygen species (ROS) and oxidative stress by its strong antioxidant potential. The molecular mechanism of protective effect of zingerone in radiation-induced apoptosis is that ROS causes Bax protein activation in apoptosis induced by radiations [27]. Accelerating expression of Bax proteins induces apoptosis, while Bcl-2 protein protects cells from apoptosis [28]. Zingerone effectively leads to the suppression of programmed cell death by decreasing apoptotic mediators including caspase activation, enhancing antiapoptotic molecules (Bcl-2) and reducing proapoptotic molecules (Bax).

## 5. Zingerone as Anticancer

Worldwide cancer is the leading cause of death representing a major public health burden. Cancer is an uncontrollable division of cells and might be incurable and may arise at any time at any age in any part of the body. It can metastasize sometimes and affect the whole body within no time. It continues to be the deadly disease that is the largest cause of mortality in the world and claims about 3500 per million lives around the world annually. Nowadays, there are various conventional anticancer therapies, but they all impart some side effects. Cancer chemoprevention has been first defined by a scientist, namely, Sporn, through the use of natural, synthetic, or biologic chemical agents that reverse, suppress, or prevent carcinogenic progression [29]. Natural dietary substances and natural pure compound including fruits, vegetables spices, flavonoids, and phenolic alkanones have drawn a great deal of attention from both the scientists and the general public owing to their ability to suppress the pathogenesis of cancers [30]. Among the natural ingredients possessing a substantial role in cancer chemoprevention is ginger. It contains some phenolic and alkanone substances which generally exert strong antioxidative, anti-inflammatory properties and potent anticarcinogenic and antimutagenic activities [31, 32]. Recent studies have shown that zingerone contains anticancer potential. It has been proved that supplementation with zingerone in DMH (dimethyl hydrazine) treated rats led to a significant decrease in tumor incidence and aberrant crypt foci formation with simultaneous modulation in the levels of tissue lipid peroxidation and antioxidant status [7].

## 6. Zingerone as Antiemetic

Among the major side effects which hamper chemotherapy in cancer patients are nausea and emesis. Though patients undergoing chemotherapies have been treated for the side effect of nausea, they still show the signs of vomiting and nausea soon after treatment regime [33]. Here, also ginger comes for the rescue and finds a role in acting as an excellent antiemetic compound. Using ginger as a supplement finds a great role in reducing cancer chemotherapy related nausea [34]. Ginger has been traditionally used as medicine all over the world since ages. It has been used to treat many gastrointestinal disorders. It acts as an effective antispasmodic. Zingerone is known to have antagonistic function against the serotonin receptors of central and peripheral nervous systems. It acts as a noncompetitive antagonist to 5-HT3 receptors in visceral afferent neurons [35]. As such it can be used to treat cytotoxic chemotherapy induced emesis and postoperative nausea and vomiting [36].

## 7. Zingerone as Lipolytic

Obesity is a complex medical condition which is related to accumulation of excess fat in the body. It has negative results on health which makes it a life threatening condition. Globally, overweight is the fifth leading cause for deaths. With obesity, likelihood for occurrence of other diseases like heart ailments and strokes, type 2 diabetes, sleep apnea, many cancers, and bone and joint disorders like osteoarthritis increase drastically. Obesity decreases the life expectancy to a great extent. Generally, obesity and overweight are caused by increased intake of energy and less physical work. Genetic susceptibility to obesity may be there but to a less extent. Hormonal imbalances, certain pharmaceutical drugs, or psychiatric illness may be some other factors for body weight increased more than normal, that is, being obese. 2.8 million adults die each year because of overweight or obesity. In addition WHO global estimates for the year 2008 reported 1.5 billion people as overweight; of these, over 200 million men and nearly 300 million women were obese [37].

Using allopathic drugs to treat obesity is in vogue [38]. These drugs generally seem to be effective, but adverse toxicities may limit their overall usefulness [39]. Antiobesity medicinal plants have wide scope to be used over synthetic drugs available [40]. Recent research in this field has suggested that herbs can treat this highly prevalent disorder to a large extent. A nutritional based treatment is being considered as an inexpensive alternative to boost weight loss and weight management [41]. Plant based medicinal plants tend to be more cost effective and above all produce markedly less or no side effects when compared to synthetic and semisynthetic drugs. Natural substances are likely to produce less toxicity and might be effective in reducing appetite. This may help in promoting significant weight loss and hence riddance from overweight and obesity [42].

Recent studies have revealed that the zingerone present in ginger has potent lipolytic activity. Workers showed that zingerone dissolves body fat, in high fat diet fed animals. Zingerone lowers blood sugar level after the oral administration of glucose in ovariectomized rats. The mechanism responsible for its lipolytic action is mediated by increasing the activity of norepinephrine sensitive lipases [43]. Zingerone has been found to be effective in enhancing basal lipolysis and isoprenaline induced lipolysis in adipocytes [44].

## 8. Conclusion

Due to changing lifestyle, the trend of using natural products for treating diseases is increasing. People all around the world are giving preference to natural products over the synthetic, due to their fewer side effects. People have become more health and nutrition conscious and use the natural drugs for pharmaceutical purposes. Ginger is a strong antioxidant, with almost insignificant drawbacks or side effects. It helps in scavenging free radicals in the body and reduces the oxidative stresses; this fundamental property of zingerone can alone help in fighting against numerous diseases of concern. It is an immunostimulant and it possesses biological actions like increasing respiratory burst, phagocytic activity, and disease resistance against pathogen. It can be used as an excellent appetizer and help in growth and maintenance of the body. There is enhanced weight gain and feed conversion efficiency. It has potent anti-inflammatory properties and thus can act as a hepatoprotective agent. It downregulates the inflammatory mediators reducing inflammation and toxicity and thus can widely be used to treat various inflammatory conditions which are the root cause of many disorders. Since many effects of zingerone have well been analysed, there is a future requirement for further studies on the clinical aspect of this natural product. Nevertheless, all the known metabolic uses of zingerone may provide novel clues for using this magnificent natural product in therapy so as to get riddance from ill effects of synthetic drugs.
